# Intestinal CD103^+^CD11b^+^ cDC2 Conventional Dendritic Cells Are Required for Primary CD4^+^ T and B Cell Responses to Soluble Flagellin

**DOI:** 10.3389/fimmu.2018.02409

**Published:** 2018-10-17

**Authors:** Adriana Flores-Langarica, Charlotte Cook, Katarzyna Müller Luda, Emma K. Persson, Jennifer L. Marshall, Nonantzin Beristain-Covarrubias, Juan Carlos Yam-Puc, Madelene Dahlgren, Jenny J. Persson, Satoshi Uematsu, Shizuo Akira, Ian R. Henderson, Bengt Johansson Lindbom, William Agace, Adam F. Cunningham

**Affiliations:** ^1^Institute of Immunology and Immunotherapy, College of Medical and Dental Sciences, University of Birmingham, Birmingham, United Kingdom; ^2^Institute of Microbiology and Infection, College of Medical and Dental Sciences, University of Birmingham, Birmingham, United Kingdom; ^3^Immunology Section, Department of Experimental Medical Science, Lund University, Lund, Sweden; ^4^VIB-Ugent Center for Inflammation Research, Ghent, Belgium; ^5^Institute of Inflammation and Ageing, College of Medical and Dental Sciences, University of Birmingham, Birmingham, United Kingdom; ^6^International Research and Development Centre for Mucosal Vaccine, Institute for Medical Science, The University of Tokyo, Tokyo, Japan; ^7^Department of Immunology and Genomics, Osaka City University Graduate School of Medicine, Osaka, Japan; ^8^World Premier International Immunology Frontier Research Centre, Osaka University, Suita, Japan; ^9^Section of Biology and Chemistry, Department for Micro- and Nanotechnology, Technical University of Denmark, Kongens Lyngby, Denmark

**Keywords:** flagellin, mucosa, immune response, dendritic cells, cDC2

## Abstract

Systemic immunization with soluble flagellin (sFliC) from *Salmonella* Typhimurium induces mucosal responses, offering potential as an adjuvant platform for vaccines. Moreover, this engagement of mucosal immunity is necessary for optimal systemic immunity, demonstrating an interaction between these two semi-autonomous immune systems. Although TLR5 and CD103^+^CD11b^+^ cDC2 contribute to this process, the relationship between these is unclear in the early activation of CD4^+^ T cells and the development of antigen-specific B cell responses. In this work, we use TLR5-deficient mice and *CD11c-cre.Irf4*^*fl*/*fl*^ mice (which have reduced numbers of cDC2, particularly intestinal CD103^+^CD11b^+^ cDCs), to address these points by studying the responses concurrently in the spleen and the mesenteric lymph nodes (MLN). We show that CD103^+^CD11b^+^ cDC2 respond rapidly and accumulate in the MLN after immunization with sFliC in a TLR5-dependent manner. Furthermore, we identify that whilst CD103^+^CD11b^+^ cDC2 are essential for the induction of primary T and B cell responses in the mucosa, they do not play such a central role for the induction of these responses in the spleen. Additionally, we show the involvement of CD103^+^CD11b^+^ cDC2 in the induction of Th2-associated responses. *CD11c-cre.Irf4*^*fl*/*fl*^ mice showed a reduced primary FliC-specific Th2-associated IgG1 responses, but enhanced Th1-associated IgG2c responses. These data expand our current understanding of the mucosal immune responses promoted by sFliC and highlights the potential of this adjuvant for vaccine usage by taking advantage of the functionality of mucosal CD103^+^CD11b^+^ cDC2.

## Introduction

The systemic and mucosal immune systems are semi-autonomous and engaging systemic immunity does not necessarily induce immunity in mucosal sites. Engaging the two immune systems concurrently could potentially enhance the benefits of vaccination, as most vaccines are administered through subcutaneous (s.c.) or intra-muscular injection. One antigen that can induce both mucosal and systemic immunity concurrently after intraperitoneal (i.p.) or s.c. immunization is purified, soluble flagellin (sFliC) from *Salmonella* Typhimurium (1–3). This 51 kDa bacterial motility protein is the only known ligand for TLR5 (4). Moreover, flagellin is an immunodominant antigen that can induce robust innate and adaptive immune responses, which can also be protective (5–7). These properties, alongside its potential as an adjuvant, mean flagellin is the focus of multiple vaccine strategies in livestock and in humans (8–12). The antigenic environment in which flagellin is encountered influences the type of immune response induced to this protein. When surface-localized on the bacterium, the antigen-specific response is Th1-reflecting, whereas to purified flagellin the response is significantly more Th2-like, including the induction of FliC-specific IgG1 (13, 14).

Conventional dendritic cells (cDC) are key initiators and modulators of adaptive immune responses and as such targeting cDC directly is an approach to enhance responses to vaccines (15, 16). cDCs can be classified into two major subsets; cDC1 that are require the transcription factors IRF8, BATF3, and ID2, and cDC2 that development is independent of these transcription factors, importantly some them require the transcription factor IRF4 for their survival and function. This classification is particularly important since it allows the identification of cDCs equivalents across tissues and even across species (17, 18). In the intestinal mucosa several sub populations of cDC can be found, CD103^+^CD11b^−^, CD103^+^CD11b^+^, and CD103^−^CD11b^+^ cDC. The first corresponds to cDC1 and the latter two to cDC2. Each of these subsets plays key, non-redundant roles in controlling immune homeostasis in the intestinal mucosa (19–21).

*In vivo* studies have shown that by 24 h after i.p. or s.c. immunization with sFliC, T cell priming is established in multiple sites concurrently, including the mesenteric lymph node (MLN), spleen and peripheral lymph nodes (1). Analysis of cDCs shows that exclusively in the MLN, there is a rapid TLR5-dependent accumulation of CD103^+^ cDC post sFliC-immunization (1). Moreover, using *Cd11c-cre.Irf4*^*fl*/*fl*^ mice, which have diminished numbers of CD103^+^CD11b^+^ cDCs in the small intestine lamina propria and a 90% reduction of this population in the MLN, we showed that this subset was essential for the induction of adaptive immune responses in the MLN, while splenic cDC2 play only a partial role. For clarity, CD103^+^CD11b^+^ cDCs will be referred to throughout as CD103^+^cDC2 (3). This indicates that i.p., immunization with sFliC can bridge both systemic and mucosal immune systems through the targeting of a single mucosal cDC subset.

Our previous work examining the role of CD103^+^cDC2 in regulating the response to sFliC focused on the long-term antibody response *in vivo* using the *Cd11c-cre.Irf4*^*fl*/*fl*^ mice. This necessitated the use of a prime-boost system and did not focus on the primary T and B cell responses. Whilst all elements of the response were lost in the MLN when mucosal CD103^+^cDC2 were reduced, some features of the anti-FliC response were retained in the spleen. This could be because some T and B cell responses were generated in the MLN shortly after immunization, which could lead to the generation of memory T and B cell responses that contribute to the responses observed after secondary immunization. Alternatively, it could be that cDC2 and cDC1 contributed differentially to the anti-sFliC response in the MLN and spleen. Therefore, we examine here the development of the anti-sFliC response in the first days after immunization to characterize the relationship between cDC2 and TLR5 and the early induction of IgG switching.

## Material and methods

### Mice

*Cd11c-cre.Irf4*^*fl*/*f*^ (19) and NAIP5^−/−^ mice were maintained at the Biomedical Center at Lund University. Specific pathogen-free 8 week C57BL/6 mice were purchased from Harlan Sprague-Dawley. TLR5^−/−^ mice were maintained in-house at the Biomedical Service Unit at the University of Birmingham. Littermates or age matched mice were used for all experiments. All animal procedures were carried out in strict accordance with the Lund/Malmö Animal Ethics Committee, the University of Birmingham Ethics Committee and were covered under the UK Home Office Project license 30/2850.

### Antigen preparation and immunization

sFliC was generated as described (22), a his-tagged recombinant protein and purified by nickel affinity chromatography and immunoprecipitation with a FliC-specific monoclonal. Mice were immunized i.p. with 20 μg recombinant sFliC for 24 h or 7 days as indicated.

### Cell isolation and flow cytometry

Single cell suspensions from spleens and MLNs were generated by mechanical disruption. When evaluating cDCs, enzymatic digestion was performed using collagenase VIII digestion (400 U/ml; 25 min; 37°C). Cells were processed for flow cytometry using previously described procedures (1). Data acquisition was performed on a LSRII (BD Bioscience) or a CyAn ADP (Beckman Coulter) and analyzed using FlowJo software 9.8.2. (Tree Star). The following FITC-conjugated antibodies were used, CD3 (145-2C11), B220 (RA3-6B2), and NK1,1 (PK136; all from eBioscience). The following PE-conjugated antibodies were used; CD103 (M290) and CD62L (MEL-14; both from eBioscience). CD11c (N418), CD44 (IM7), and CD95 (MFL3; all from eBioscience) were PE-Cy7-conjugated. CD11b (M1/70), CD4 (RM4-5, both BD Biosciences), TCRβ (H57-597, Biolegend), GL7 (eBioscience) were PB-conjugated. MHC-II (M5/114.15.2) and Streptavidin (eBioscience) APC-conjugated where used. B220 (RA3-6B2), CD11b (MI-70), CD11c (N418) and NK1.1 (PK136; all from eBioscience) were Alexa700-conjugated. TCRβ (H57-597, Biolegend) was APC Cy7-conjugated. CD8α (5H10) from Invitrogen was used PO-conjugated.

cDCs were gated as Lin^−^[CD3,B220,NK1.1,GR1]CD11c^+^MHC^hi^ cells, splenic cDC1 were defined as CD8α^+^ and cDC2 as CD11b^+^CD4^+^ cells, mucosal cDC1 were defined as CD103^+^CD11b^−^ and cDC2 as CD103^+^CD11b^+^ cells. Activated CD4^+^ T cells were gated as CD3^+^CD4^+^CD44^+^CD62L^−^. Germinal center (GC) B cells were defined as TCRβ^−^CD19^+^GL7^+^CD95^+^ cells.

### Immunohistochemistry and confocal microscopy

Immunohistochemistry was performed as described previously (13). Cryosections (6 μm) were incubated with primary unlabelled Abs for 45 min at RT before addition of either HRP-conjugated or biotin-conjugated secondary antibodies. FliC-binding cells were identified as described (1) using soluble biotinylated FliC. Subsequently streptavidin ABComplex alkaline phosphatase (Dako) was used. Signal was detected using diaminobenzidine for HRP activity and naphthol AS-MX phosphate with Fast Blue salt and levamisole for alkaline phosphatase activity. Images were acquired using a Leica microscope DM6000 using 10x and 20x objectives. Quantification of sFliC^+^IgG1^+^ and sFliC^+^IgG2c^+^ was performed in two independent experiments, each with 4 mice per group. A total of 10 random fields were evaluated per slide.

Immunofluorescence was performed on frozen sections. Staining was performed in PBS containing 10% FCS, 0.1% sodium azide and sections were mounted in 2.5% 1,4-Diazabicyclo(2,2,2)octane (pH 8.6) in 90% glycerol in PBS. After incubation with primary Abs (1 h, room temperature), secondary Abs were added (30 min; room temperature). Images were acquired using the Fluorescence Zeiss Axio Scan.Z1, image analysis was performed with Zen, 2012 blue edition.

The following Abs were used for immunofluorescence: CD11c, Dec205, CD103. DCIR2 was used biotinylated. CD11b (eBioscience) and streptavidin (Jackson Immunoresearch) were used A488 conjugated. And AMCA-conjugated anti-IgM (Jackson Immunoresearch) was used. The following Abs were used for immunohistochemistry: IgD, IgG1, and IgG2c. sFliC and PNA (Vector) biotinylated were used.

### *In vitro* restimulation for the detection of antigen-specific CD4^+^ T cells

Antigen-specific CD4^+^ T cells were detected by their expression of CD154 post-restimulation as previously described (23). In brief, 6 × 10^6^ cells (from spleen or MLN) were cultured in the presence of sFliC (5 μg/ml), anti-CD40 (1C10 2 μg/ml), biotinylated anti-CD154 (MR1 5 μg/ml) and anti-Fcγ receptor (2.4G2 50 μg/ml) for 48 h. Control wells included cells cultured without antigen. The expression of CD154 was evaluated by staining the cells with Streptavidin-APC and gating was done on Lin^−^(CD19/CD11b/NK1.1/CD11c)TCRβ^+^CD4^+^CD62L^−^.

### Sflic-specific ELISA

ELISA plates were coated with 5 μg/ml of sFliC (24 h at 4°C) and blocked with 1% BSA overnight at 4°C. Serum was diluted 1:100 in PBS−0.05% Tween, and was further diluted stepwise. Plates were incubated for 1 h at 37°C. Bound antibodies were detected using alkaline phosphatase conjugated, goat anti-mouse IgG, IgG1, and IgG2c Abs (Southern Biotech). Alkaline phosphatase activity was detected using Sigma-Fast p-nitrophenylphosphate (Sigma Aldrich). Relative reciprocal titers were calculated by measuring the dilution at which the serum reached a defined OD_405_.

### Statistics

For statistics we used the non-parametric Mann-Whitney sum of ranks test or two-way ANOVA as appropriate using the GraphPad Prism software (GraphPad).

## Results

### The accumulation of CD103^+^cDC2 in the MLN after sFLIC immunization is TLR5-dependent

**Figure 1:**
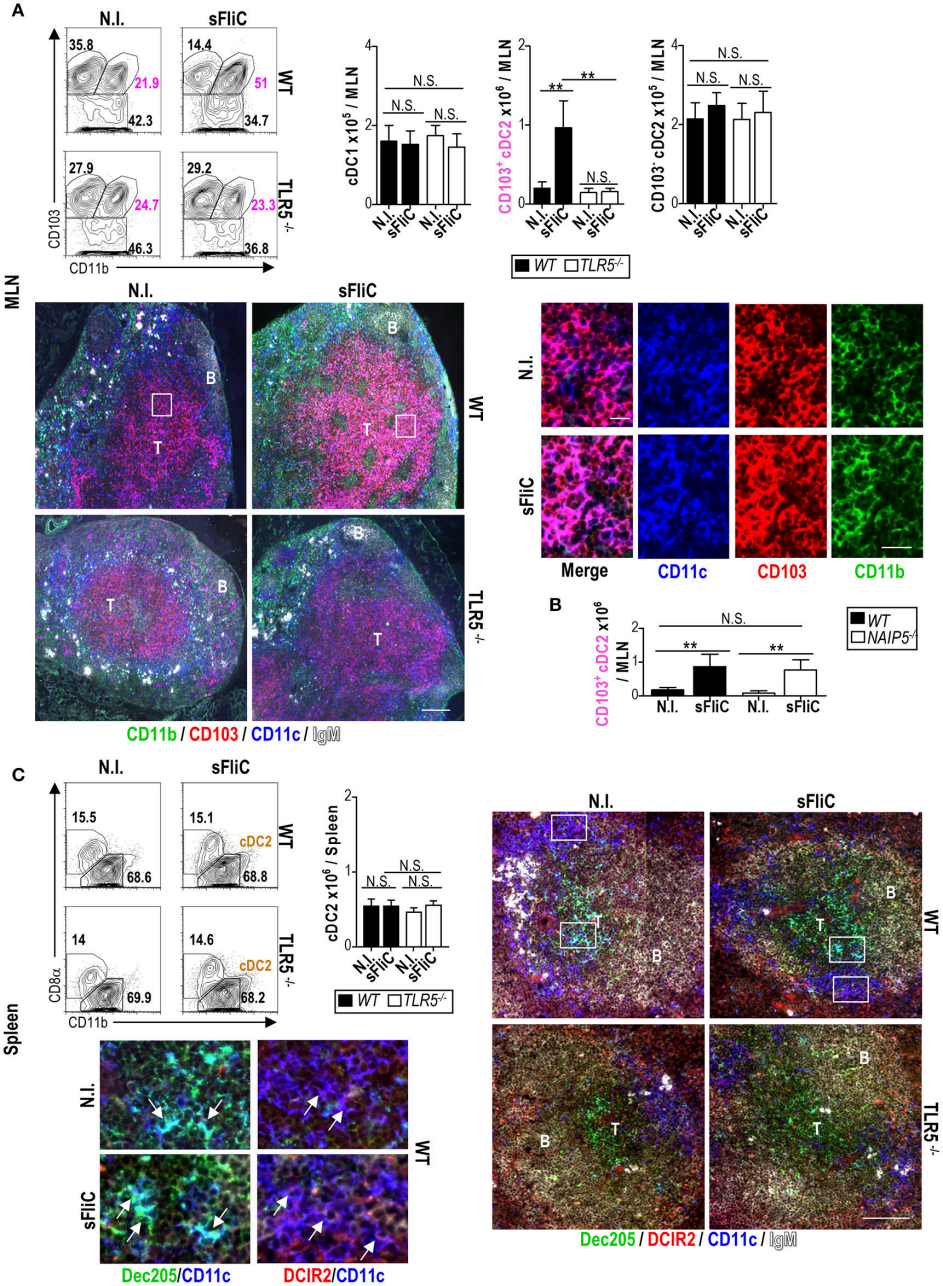
Mucosal CD103^+^cDC2 respond to sFliC immunization in a TLR5-dependent manner. Wild-type (WT) or TLR5^−/−^ mice were immunized i.p. with sFliC and cDCs (Lin^−^MHC-II^hi^CD11c^hi^) were evaluated 24 h later, alongside non-immunized (N.I.) mice. **(A)** MLN representative flow cytometry plots (including percentages) of cDC1 (Lin^−^MHC-II^hi^CD11c^hi^CD11b^−^CD103^+^), CD103^+^cDC2s (Lin^−^MHC-II^hi^CD11c^hi^CD11b^+^CD103^+^) and CD103^−^cDC2 (Lin^−^MHC-II^hi^CD11c^hi^CD103^−^) are shown with adjacent graphs of absolute numbers. Representative photomicrographs of MLN sections stained for CD11c; blue, CD103; red, CD11b; green, and IgM; white (scale bar = 200 μm) are shown (top right). Zoom-in insets (white boxes) show single staining and a merge of CD11c, CD103, and CD11b (scale bar = 20 μm). T, T zone; B, B zone. **(B)** WT or NAIP5^−/−^ mice were immunized i.p., with sFliC and absolute numbers of MLN CD103^+^cDC2s were evaluated 24 h later, alongside non-immunized (N.I.) mice. **(C)** Wild-type (WT) or TLR5^−/−^ mice were immunized i.p. with sFliC and splenic cDCs (Lin^−^MHC-II^hi^CD11c^hi^) were evaluated 24 h later, alongside non-immunized (N.I.) mice. Representative flow cytometry plots (including percentages) of cDC2s (Lin^−^MHC-II^hi^CD11c^hi^CD11b^+^) are shown with adjacent graphs of absolute numbers. Representative photomicrographs of spleen sections stained for CD11c; blue, Dec205; green, DCIR2; red, and IgM; white (scale bar = 100 μm). Zoom-in insets (white boxes) show the differential location of cDC1s (Dec205^+^) in the T zone and cDC2s (DCIR2^+^) in the bridging channels. Data shown as mean+s.d. of 4 mice and are representative of 3 independent experiments. **P < 0.001, by two-way analysis of variance (ANOVA) N.S., not significant.

Intraperitoneal immunization of sFliC induces a rapid MyD88-dependent accumulation of intestinal-derived CD103^+^cDC2 in the MLN (1, 3). To analyse the role of TLR5 in this response, WT and TLR5^−/−^ mice were immunized with sFliC and the cDC response analyzed by flow cytometry and *in situ* by immunofluorescence 24 h post-immunization. In the MLN of WT mice, immunization with sFliC resulted in an increase in the frequency and absolute numbers of CD103^+^cDC2, which was abrogated in TLR5^−/−^ mice, no significant change in absolute numbers or frequency was observed for cDC1 or CD103^−^cDC2 (Figure 1A). Immunofluorescence microscopy showed that CD103^+^cDC2 were mainly located in the T zone, before and after immunization, although they were less abundant in the absence of immunization. The high zoom-insets confirmed that CD103^+^cDC2 in the T zone express CD11c, CD103, and CD11b. TLR5 is the extracellular receptor for sFliC, however there is an intracellular pathway for flagellin detection controlled by NAIP5 (24). To evaluate the contribution of this alternative pathway we evaluated the CD103^+^cDC2 in NAIP5^−/−^ mice, we observed a similar accumulation of those cells in comparison with the WT after sFliC immunization, strongly suggesting that TLR5 exclusively controls the response to sFliC by CD103^+^cDC2 in the MLN (Figure 1B).

In contrast to the MLN, in the spleen there was no change in the frequency and absolute numbers of cDC2 (identified as CD11b^+^ cDC) of WT mice after immunization with sFliC (Figure 1C). Furthermore, no difference was observed in the location of cDC2 (DCIR2^+^) which remain primarily localized in the bridging channels between the red and white pulps (25). Loss of TLR5 did not alter the numbers of cDC2 before or after immunization, nor the distribution of these cells within the spleen (Figure 1B). Therefore, immunization with sFliC results in the selective accumulation of CD103^+^cDC2 in the MLN, but not in the spleen in a TLR5 dependent manner.

### CD103^+^cDC2 are essential for T cell priming in the MLN after immunization with sFLIC

**Figure 2:**
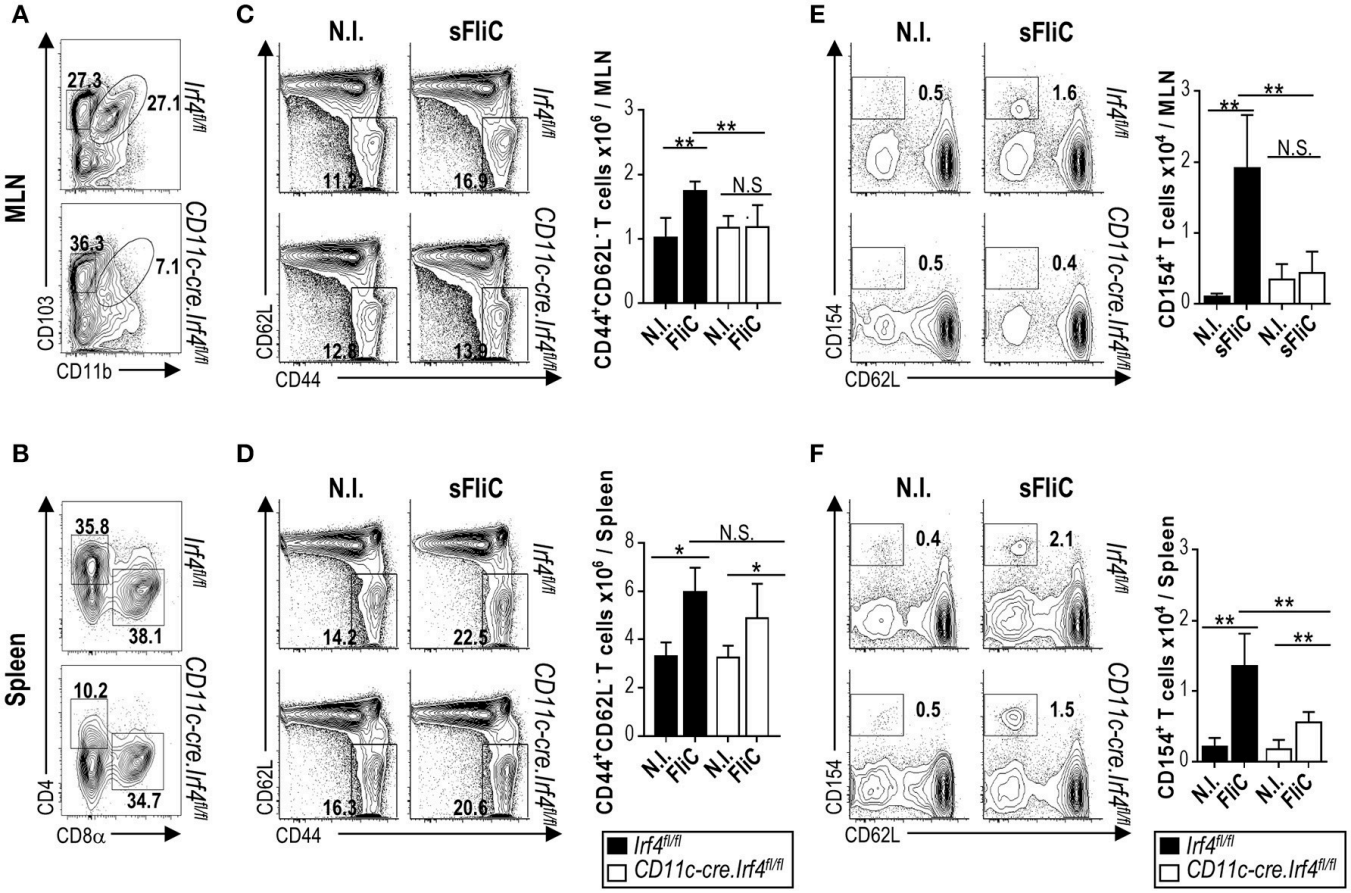
The primary T cell response to sFliC is dependent upon CD103^+^cDC2 in the mucosa but not in the spleen. cDC populations in *Irf4*^*fl*/*fl*^ or *Cd11c-cre.Irf4*^*fl*/*fl*^ mice were evaluated in (**A)** MLN and **(B)** spleen. *Irf4*^*fl*/*fl*^ or *Cd11c-cre.Irf4*^*fl*/*fl*^ mice were either non-immunized (N.I.) or immunized with sFliC and T cell responses were evaluated 7 days later. Representative flow cytometry plots (percentages) and absolute numbers (graphs) of activated CD4^+^ T cells (CD3^+^CD4^+^CD44^+^CD62L^−^) in the **(C)** MLN and **(D)** spleen. *Ex vivo* sFliC-specific restimulations, single cell suspensions were restimulated with 5 μg/ml of sFliC in the presence of anti-CD40 (2 μg/ml), biotinylated anti-CD154 (5 μg/ml) for 48 h. Antigen-specific CD4^+^ T cells were identified by detection of the anti-CD154 by streptavidin. **(E)** MLN and **(F)** spleen representative flow cytometry plots (percentages) and absolute numbers (graphs) of CD154^+^ CD4^+^ T cells. Data shown as mean + s.d. (*n* = 4 mice/group) representative experiment of 3 performed. **P < 0.001; *P < 0.05, by two-way analysis of variance (ANOVA), N.S., not significant.

To assess the contribution of cDC2 to T cell priming after immunization with sFliC, we assessed responses in *Cd11c-cre.Irf4*^*fl*/*fl*^ mice. These mice lack IRF4 in cells that express CD11c, resulting in a 50% reduction of CD103^+^CD11b^+^ cDCs in the small intestine lamina propria and a 90% reduction in the MLN (19). Figure 2A shows the significant reduction of MLN CD103^+^CD11b^+^ cDCs in *Cd11c-cre.Irf4*^*fl*/*fl*^ mice in comparison with the *Irf4*^*fl*/*fl*^ mice. Furthermore, we also show the expected reduction of splenic cDC2 in these mice (Figure 2B). The frequency and number of activated CD4^+^ T cells in the MLN of *Cd11c-cre.Irf4*^*fl*/*fl*^ mice 7 days after FliC immunization, was lower compared to *Irf4*^*fl*/*fl*^ mice and similar to non-immunized mice (Figure 2C). In contrast, absolute numbers of activated CD4^+^ T cells in the spleen were similar between *Cd11c-cre.Irf4*^*fl*/*fl*^ and *Irf4*^*fl*/*fl*^ mice (Figure 2D). To examine the endogenous antigen-specific T cell response we performed an *in vitro* re-stimulation essay that uses the transient expression of CD154 to identify antigen-specific CD4^+^ T cells (23). In the MLN from immunized *Cd11c-cre.Irf4*^*fl*/*fl*^ mice, the frequency and number of CD154^+^ sFliC-specific CD4^+^ T cells was significantly lower than those in immunized *Irf4*^*fl*/*fl*^ mice and similar to levels observed in from non-immunized mice (Figure 2E). In contrast, in the spleen an increase of CD154^+^ sFliC-specific CD4^+^ T cells was observed in the *Cd11c-cre.Irf4*^*fl*/*fl*^ mice compared to the non-immunized mice but it was significantly lower compared to *Irf4*^*fl*/*fl*^ mice (Figure 2F). Collectively, these results demonstrate that the early T cell response induced to sFliC is dependent on mucosal CD103^+^cDC2s, however in the spleen IRF4 expression by cDCs only impacts partially on T cell priming.

### cDC2 influence the extent and direction of IgG switching

**Figure 3:**
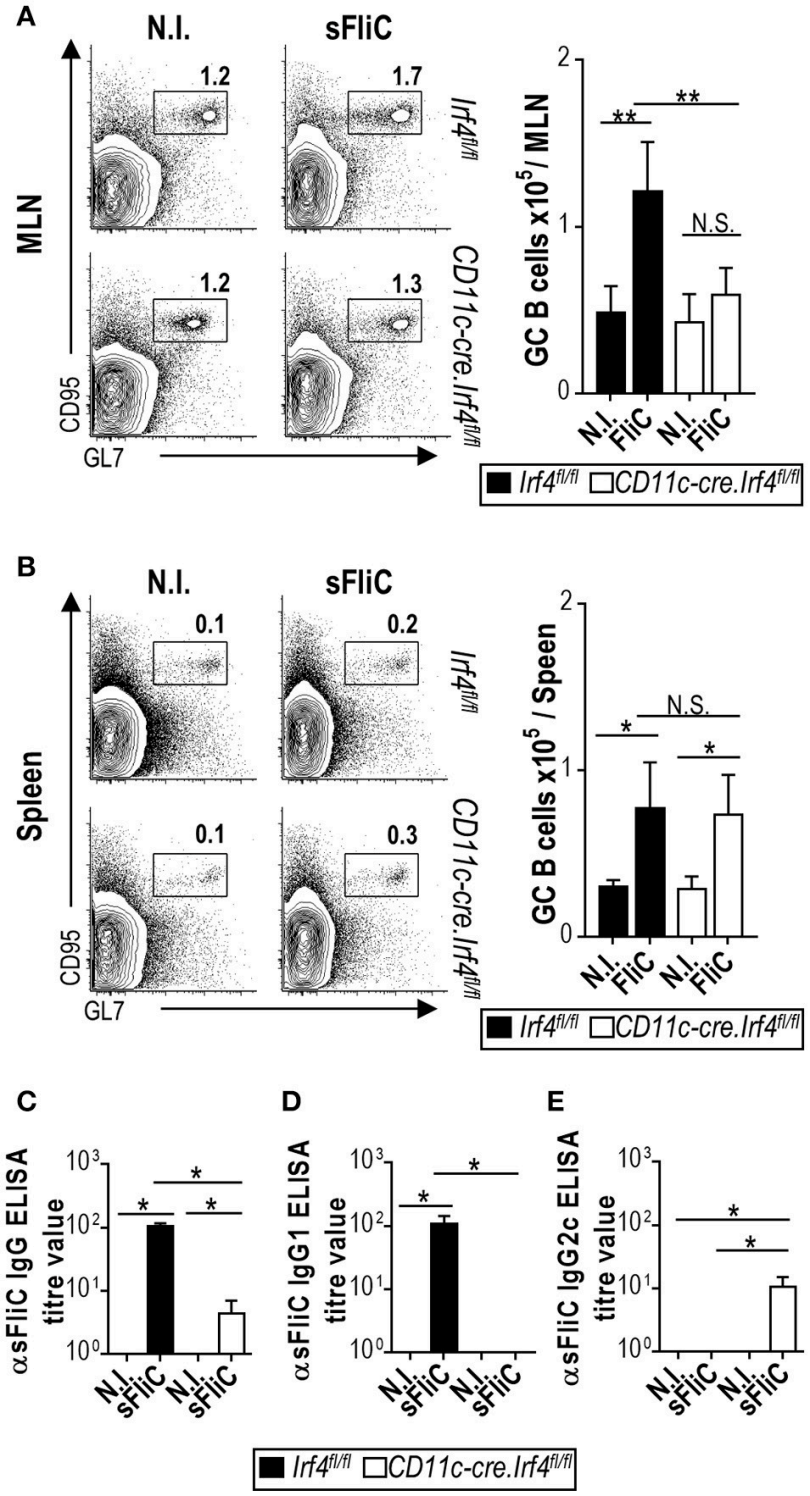
The generation of primary B cell responses to sFliC in the MLN, but not the spleen, are dependent upon CD103^+^cDC2. *Irf4*^*fl*/*fl*^ or *Cd11c-cre.Irf4*^*fl*/*fl*^ mice were either non-immunized (N.I.) sFliC-immunized and GC B cells (TCRβ^−^CD19^+^GL7^+^CD95^+^) were evaluated 7 days later. **(A)** MLN and **(B)** spleen representative flow cytometry plots (percentages) and absolute numbers (graphs) of GC B cells. Data are mean+s.d. (*n* = 4 mice/group) representative experiment of 3 performed. **P < 0.001; *P < 0.05, by two-way analysis of variance (ANOVA), NS, not significant. **(C)** Serum anti-sFliC IgG, IgG1, and IgG2c evaluated by enzyme-linked immunosorbent assay (ELISA). Data shown as mean + s.d. (*n* = 12 mice/group) and shows three independent experiments pooled together. *P < 0.05, two-way analysis of variance (ANOVA), N.S., not significant.

In order to address how the B cell response to sFliC is affected in the *Cd11c-cre.Irf4*^*fl*/*fl*^ mice we analyzed GC B cells by flow cytometry 7-days post-immunization. In the MLN, *Cd11c-cre.Irf4*^*fl*/*fl*^ mice showed no increase in the number of GC B cells in comparison to non-immunized and *Irf4*^*fl*/*fl*^ sFliC-immunized mice (Figure 3A). In contrast, in the spleen, immunized *Cd11c-cre.Irf4*^*fl*/*fl*^ and *Irf4*^*fl*/*fl*^ mice displayed a similar increase in GC B cell numbers (Figure 3B). To analyse the FliC-specific Ab response in more detail, serum Ab titres were evaluated by ELISA. Total sFliC-specific IgG titers were reduced in *Cd11c-cre.Irf4*^*fl*/*fl*^ mice in comparison with *Irf4*^*fl*/*fl*^ mice (Figure 3C). In mice, sFliC induces some Th2-associated features including antigen-specific IgG1 (13, 14). FliC-specific IgG1 was detected in sFliC-immunized *Irf4*^*fl*/*fl*^ mice, but was absent in *Cd11c-cre.Irf4*^*fl*/*fl*^ mice (Figure 3D). This was unexpected since some sFliC-specific IgG was detected in *Cd11c-cre.Irf4*^*fl*/*fl*^ mice and so titers of Th1-associated IgG2c were assessed. This isotype was detected exclusively in sFliC-immunized *Cd11c-cre.Irf4*^*fl*/*fl*^ mice (Figure 3E), suggesting that cDC2 contribute to IgG1 switching.

**Figure 4:**
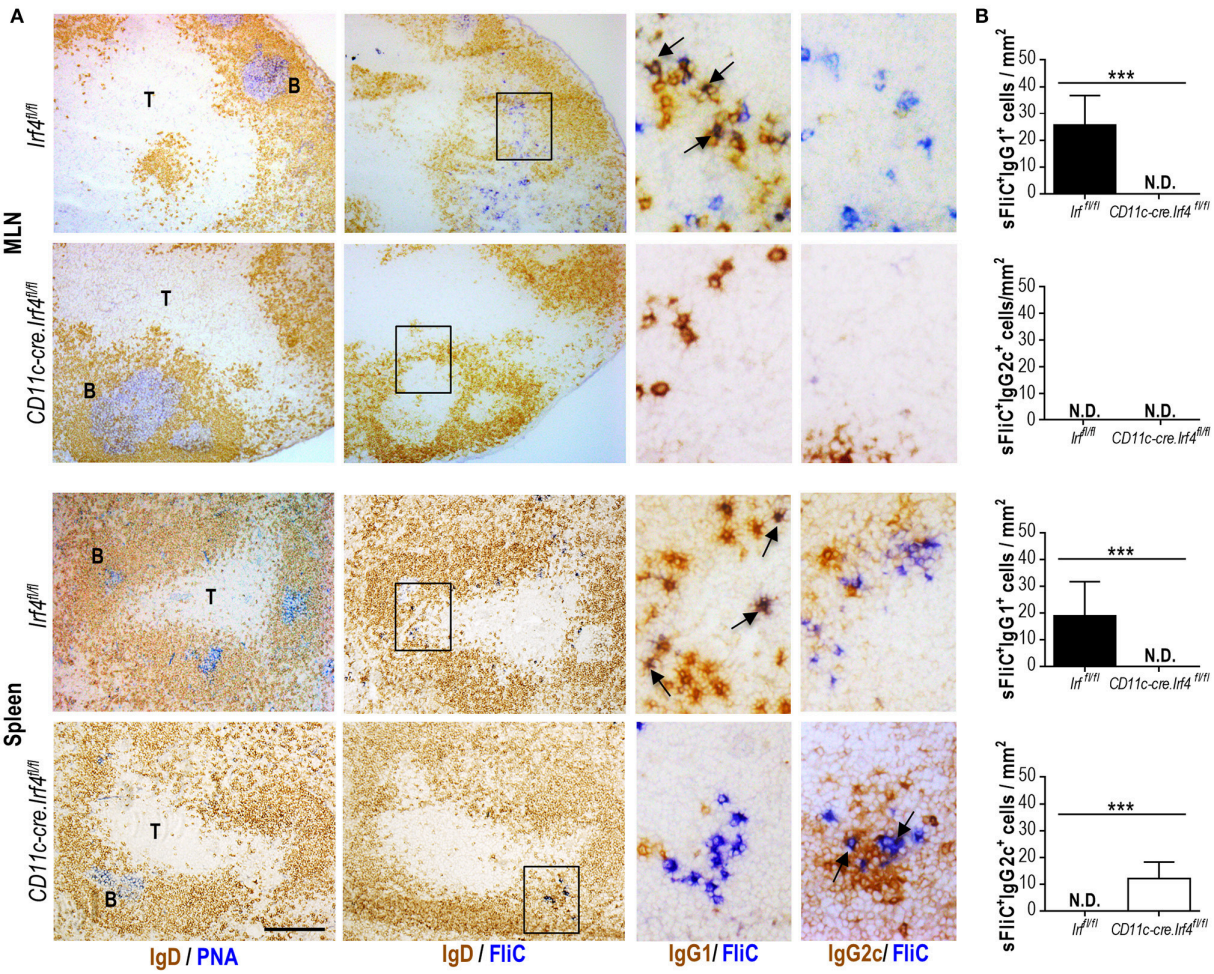
Switching to IgG1 is abrogated in the *CD11c-cre.Irf4*^*fl*/*fl*^ mice. *Irf4*^*fl*/*fl*^ or *Cd11c-cre.Irf4*^*fl*/*fl*^ mice were sFliC-immunized for 7 days. **(A)** Representative photomicrographs of serial sections from MLN and spleen stained for: PNA-binding cells (blue) and IgD-expressing cells (brown) (first column) or sFliC-binding cells (blue) and IgD-expressing cells (brown) (second column), scale bar = 200 μm. The third and fourth columns show zoom-in insets (black-boxed areas) stained to detect sFliC-binding cells and IgG1 and IgG2c respectively (scale bar = 50 μm). T, T zone; B, B zone. **(B)** Quantification of sFliC^+^IgG1^+^ cells and sFliC^+^IgG2c^+^ cells in the MLN and spleen. A total of 10 random fields were evaluated per slide. Data shown as mean + s.d. (*n* = 8 mice pooled from two independent experiments). ***P < 0.0001, by Mann-Whitney. N.D. non-detected.

We hypothesized that the serum IgG2c derived from spleen. To analyze this possibility *in situ*, we performed immunohistochemistry on serial sections from the MLN and spleen of sFliC-immunized *Irf4*^*fl*/*fl*^ and *Cd11c-cre.Irf4*^*fl*/*fl*^ mice. GCs were identified as follicular areas that bind PNA and sFliC-specific cells were identified by using biotinylated sFliC in conjunction with either anti-IgG1 or anti-IgG2c Abs. In the MLN, sFliC-specific cells were exclusively observed in *Irf4*^*fl*/*fl*^ mice and were IgG1^+^ (Figure 4A). In contrast, sFliC-binding cells were found in the spleens of both *Irf4*^*fl*/*fl*^ and *Cd11c-cre.Irf4*^*fl*/*fl*^ mice whilst in the *Irf4*^*fl*/*fl*^ mice, the sFliC-specific cells were IgG1^+^ and IgG2c^−^. In contrast, in *Cd11c-cre.Irf4*^*fl*/*fl*^ mice the sFliC-specific cells were exclusively IgG2c^+^ (Figure 4B). Collectively, these results show that cDC2s play a role in the polarization of the Ab response *in vivo*.

## Discussion

We have previously shown that sFliC can drive a long-term mucosal adaptive response after i.p. immunization (1, 3). Furthermore, we also have shown that s.c. and i.p. immunization both induce similar cDC and IgA responses in the MLN, suggesting that our observations are not dependent of the route of immunization, but due to the intrinsic properties of sFliC. When addressing the prime-boost immune response in *Cd11c-cre.Irf4*^*fl*/*fl*^ mice the immune response to sFliC in the spleen was reduced, but not abrogated, suggesting the possibility that memory cells could contribute to the response. To address this possibility we studied the primary immune response to sFliC and show that the primary T and B cell responses to sFliC in the MLN are completely dependent on CD103^+^cDC2, while that in the spleen is only partially dependent on cDC2.

Mucosal CD103^+^cDC2s are probably more efficient at driving responses after sFliC immunization because of their high expression levels of TLR5 in comparison to splenic cDCs (26, 27). TLR5 can play an additional role in enhancing antigen capture and presentation through MHC-II and this is not MyD88-dependent (28). Moreover, ligation of TLR5 itself will lead to an upregulation of co-stimulatory molecules and cytokine expression, in a MyD88-dependent manner (4, 14). This is likely to be the mechanism that mediates the accumulation of CD103^+^cDC2 into the MLN after immunization with sFliC (1, 3). Furthermore, splenic cDCs are able to respond rapidly after sFliC immunization, possibly through activation by a bystander effect (22, 29). Therefore, in the spleen there may be less of a selective advantage for one subset to capture sFliC over another, meaning that both cDC1 and cDC2 are potentially able to present antigen to CD4^+^ T cells and initiate priming. In support of this idea *ex vivo* data using sorted, *in vivo* loaded, cDCs showed that both splenic cDC1 and cDC2 are able to mediate to T cell priming. Additionally, when flagellin is used as an adjuvant in studies using DEC205 and 33D1 to target splenic cDC1 and cDC2 cells, it shows that the presence of flagellin enhances the capacity of both cDC subsets to mediate T cell proliferation (30). This demonstrates that flagellin can promote responses in both cDC subsets, which indirectly supports our findings. In contrast to this, in the intestinal mucosa only CD103^+^cDC2 mediate T cell priming (3). Therefore, despite having a similar ontogeny, mucosal CD103^+^cDC2 and splenic cDC2 show differences in their capacity to capture sFliC and this difference may account for why cDC2 play such a dominant role in driving T cell responses to FliC in the MLN but not the spleen.

After primary immunization of WT mice with sFliC, there is a robust GC response in the spleen, but a limited extrafollicular plasma cell response (13, 22). The predominant antigen-specific IgG isotype detected after immunization with sFliC in the serum is IgG1, associated to a Th2-like response (13, 14). Importantly, in the *Cd11c-cre.Irf4*^*fl*/*fl*^ mice there was no significant increase in numbers of GC B cells in the MLN. In contrast, in the spleen, there was a normal GC response, which suggested that a B cell response developed in the spleen but not in the MLN when CD103^+^cDC2 were reduced. Nevertheless, there were lower serum total IgG titers in the *CD11-cre.Irf4*^*fl*/*fl*^ mice and an abrogated IgG response in the BM (3). One interpretation of this is that in the primary response to sFliC, the antibody response generated in the MLN is a significant, if not predominant, contributor, to the serum total FliC-specific IgG pool.

A more detailed analysis of the IgG response showed that there was a difference in the predominant IgG isotype induced between immunized *Cd11c-cre.Irf4*^*fl*/*fl*^ and *Irf4*^*fl*/*fl*^ mice. Inducing the appropriate IgG isotype is important as the distinct IgG isotypes can influence the level of protection afforded by vaccination (31) or against different pathogens (32). Surprisingly, the residual FliC-specific IgG response observed in *Cd11c-cre.Irf4*^*fl*/*fl*^ mice was not of the IgG1 isotype, but instead was of the IgG2c isotype. Immunohistochemistry showed that the FliC-specific IgG2c was being produced locally in the spleen by cells proximal to GC (Figure 3). This suggests that although a B cell response is maintained in the spleens of the *Cd11c-cre.Irf4*^*fl*/*fl*^ mice, this B cell response is substantially different qualitatively. Further work is needed to identify what B cell associated factors, such as BAFF or APRIL, cDC1 and cDC2 produce that may contribute to this and whether these differ between cDCs from different anatomical sites. In mice, there is a partial association between the direction of the T helper response and IgG isotype switching and so these findings may suggest that cDC2 contribute to Th2-associated responses. An association between cDC2 and Th2 polarization has been described previously in the context of infection or in atopic asthma models (21, 33–35). Moreover, we are working toward developing strategies to conjugate sFliC to different antigens and evaluate if these features that sFliC is able to promote as an adjuvant can be transferred to clinically relevant antigens. Our data helps inform on the relative merits of targeting specific DC populations in vaccination strategies.

## Author contributions

Conceptualization: AF-L, AC, and WA. Methodology: AF-L, CC, NB-C, JY-P, IH, MD, and BL. Investigation: AF-L, KM, EP, JM, NB-C, and JY-P. Resources: JP, SU, and SA. Writing—original draft: AF-L. Writing, review, and editing: AF-L, AC, and WA. Funding acquisition: AF-L, AC, and WA.

### Conflict of interest statement

The authors declare that the research was conducted in the absence of any commercial or financial relationships that could be construed as a potential conflict of interest.

## Abbreviations

Ab: antibody
cDCs: conventional dendritic cells
GC: germinal center
i. p.: intraperitoneal
MLN: mesenteric lymph node
s.c.: subcutaneous
sFliC: soluble flagellin.
